# Evaluation of patients with a HeartMate 3 left ventricular assist device using echocardiographic particle image velocimetry

**DOI:** 10.1007/s40477-020-00533-z

**Published:** 2020-11-26

**Authors:** Arend F. L. Schinkel, Sakir Akin, Mihai Strachinaru, Rahatullah Muslem, Dan Bowen, Yunus C. Yalcin, Jasper J. Brugts, Alina A. Constantinescu, Olivier C. Manintveld, Kadir Caliskan

**Affiliations:** 1 Department of Cardiology, Thoraxcenter, Erasmus MC, Room Rg427, ‘s-Gravendijkwal 230, 3015 CE Rotterdam, The Netherlands; 2 Department of Intensive Care, Haga Teaching Hospital, The Hague, The Netherlands

**Keywords:** Cardiomyopathy, HeartMate 3, Left ventricular assist device, Particle image velocimetry

## Abstract

**Purpose:**

Poor left ventricular (LV) function may affect the physiological intraventricular blood flow and physiological vortex formation. The aim of this study was to investigate the pattern of intraventricular blood flow dynamics in patients with LV assist devices (LVADs) using echocardiographic particle image velocimetry.

**Materials and methods:**

This prospective study included 17 patients (mean age 57 ± 11 years, 82% male) who had received an LVAD (HeartMate 3, Abbott Laboratories, Chicago, Illinois, USA) because of end-stage heart failure and poor LV function. Eleven (64%) patients had ischemic cardiomyopathy, and six patients (36%) had nonischemic cardiomyopathy. All patients underwent echocardiography, including intravenous administration of an ultrasound-enhancing agent (SonoVue, Bracco, Milan, Italy). Echocardiographic particle image velocimetry was used to quantify LV blood flow dynamics, including vortex formation (Hyperflow software, Tomtec imaging systems Gmbh, Unterschleissheim, Germany).

**Results:**

Contrast-enhanced ultrasound was well tolerated in all patients and was performed without adverse reactions or side effects. The LVAD function parameters did not change during or after the ultrasound examination. The LVAD flow was on average 4.3 ± 0.3 L/min, and the speed was 5247 ± 109 rotations/min. The quantification of LV intraventricular flow demonstrated substantial impairment of vortex parameters. The energy dissipation, vorticity, and kinetic energy fluctuation indices were severely impaired.

**Conclusions:**

Echo particle velocimetry is safe and feasible for the quantitative assessment of intraventricular flow in patients with an LVAD. The intraventricular LV flow and vortex parameters are severely impaired in these patients.

## Introduction

Patients with end-stage heart failure and poor left ventricular (LV) function due to ischemic or nonischemic cardiomyopathy may benefit from therapy with an LV assist device (LVAD). These devices may significantly improve patients’ quality of life, alleviate symptoms, and prolong life, but there are concerns about complications, including thrombosis, stroke, and device durability [1–3]. Some of these LVAD-related complications are possibly a result of impaired physiological blood flow dynamics.

Echocardiographic particle image velocimetry combined with an ultrasound-enhancing agent is a novel method for quantitatively assessing intraventricular blood flow dynamics [4]. The ultrasound-enhancing agent consists of microbubbles, which have a size comparable to that of erythrocytes and which serve as a tracer of intraventricular blood flow [5, 6]. Recently, this method has successfully been explored in experimental and clinical research settings [7–11]. In a normal heart, the size and shape of the LV, together with the orientation of the valves, favor vortex formation and spontaneous redirection of the blood flow from the LV inflow to the LV outflow tract [4]. The physiological vortex formation is supposed to be an energy-efficient arrangement to avoid excessive wall stress and optimize LV pump performance. Presumably, poor LV function, in addition to pathological alterations in heart volume and shape in patients with ischemic or nonischemic cardiomyopathy, affects intraventricular blood flow dynamics and vortex formation. Moreover, in patients with advanced heart failure due to poor LV function who receive LVAD therapy, the inflow cannula located near the LV apex and the continuous suction of the device may affect blood flow dynamics and vortex formation.

The ultimate goal of echo particle velocimetry in patients with an LVAD is to obtain an improved understanding of LV blood flow dynamics, to optimize treatment, and to reduce the number of blood-flow-related complications. The aim of this study was to investigate the pattern of intraventricular blood flow dynamics in patients with an LVAD using echocardiographic particle image velocimetry. The degree of blood flow disturbance was quantified by energy dissipation, vorticity, and kinetic energy fluctuation indices.

## Methods

Consecutive ambulatory patients who had received an LVAD (HeartMate 3, Abbott Laboratories, Chicago, Illinois, USA) because of end-stage heart failure due to ischemic or nonischemic cardiomyopathy and poor LV function were asked to participate in this prospective study. The study protocol was approved by the Medical Ethics Committee of the Erasmus Medical Center, Rotterdam, The Netherlands. All patients provided a signed informed consent form. All patients underwent transthoracic echocardiography using an ultrasound-enhancing agent. Exclusion criteria were contraindications for the use of an ultrasound-enhancing agent, such as unstable angina, acute cardiac failure, acute endocarditis, and known allergies for ultrasound contrast agents.

Transthoracic echocardiography was performed with a Philips Epiq 7C ultrasound system (Philips Medical Systems, Bothell, USA), using an X5-1 transducer. A standardized image acquisition protocol based on the American Society of Echocardiography guidelines was used [12]. Parasternal long-axis and short-axis views, as well as apical four-, two-, and three-chamber views, were obtained using B-mode ultrasound and color Doppler imaging.

Contrast-enhanced echocardiography was performed using the contrast mode of the ultrasound system, which employs amplitude modulation techniques and a mechanical index of 0.1–0.5 to optimize the images. The sector width and depth were optimized to obtain complete LV views and to maximize the frame rate. The ultrasound-enhancing agent (SonoVue™, sulfur hexafluoride microbubble suspension, Bracco S.p.A., Milan, Italy) was administered intravenously in boluses of 0.5 mL. Image acquisition was performed during the dilution phase, which follows the peak concentration of the ultrasound-enhancing agent, when the LV was homogeneously filled for an optimal visualization of blood flow, while avoiding both saturation and black areas [4]. The bolus administration was repeated when necessary up to a total dose of 5.0 mL. During and after administration of the ultrasound-enhancing agent, patients were observed for potential side effects or complications, and LVAD function parameters were monitored. For both standard and contrast echocardiographies, cine clips were digitally stored and reviewed offline. The standard and contrast echocardiographic studies were reviewed offline by two observers unaware of the clinical data. Quantitative analysis of LV blood flow dynamics was performed offline using Hyperflow software (Tomtec imaging systems Gmbh, Unterschleissheim, Germany). This software package was previously validated and has good accuracy for the assessment of intraventricular flow and flow direction [9, 10]. The software does not depend on anthropometric parameters. Electrocardiographic tracing was used to confirm the systolic and diastolic phases. The vortex calculations were performed in the apical three-chamber view. Vortex parameters were computed from the steady-streaming vortex, including the vortex area normalized to the LV area, the vortex intensity (the integral of the vorticity inside the vortex) normalized to the total vorticity, the vortex depth (the distance from the vortex center to the vortex base), and the vortex length along the base–apex direction, both normalized to the LV length.

Statistical analyses were performed using SPSS for Windows (version 17.0, SPSS, Chicago, USA) and Excel (Excel 2003, Microsoft, Redmont, USA). Continuous variables are reported as mean ± standard deviation. Categorical variables are expressed as percentages. The Chi-square test was used to evaluate differences between proportions. A *p* value < 0.05 was considered to indicate a statistically significant difference.

## Results

The baseline characteristics of the study population (mean age 57 ± 11 years, 82% male) are presented in Table 1. Eleven (65%) patients had received an LVAD because of ischemic cardiomyopathy, and six patients (35%) had nonischemic cardiomyopathy.

**Table 1:** Clinical characteristics of the study population

Characteristic	Data
Age (years)	57 ± 11
Men	14 (82)
Height (cm)	179 ± 8
Weight (kg)	80 ± 12
BMI (kg/m^2^)	25 ± 4
Heart rate (beats/min) Systolic blood pressure (mmHg) Diastolic blood pressure (mmHg) NYHA class 3	80 ± 12 100 ± 6 61 ± 7 5 (29)
NYHA class 4	12 (71)
Ischemic cardiomyopathy	11 (65)
Nonischemic cardiomyopathy	6 (35)
Previous paroxysmal atrial fibrillation	7 (41)
Previous ventricular tachycardia	11 (65)
Percutaneous coronary intervention	7 (41)
Coronary bypass surgery	4 (24)

Data are presented as numbers of patients (percentages) or as mean ± standard deviation

*BMI* Body mass index, *NYHA* New York Heart Association

Contrast-enhanced ultrasound was well tolerated in all patients and was performed without adverse reactions or side effects. The LVAD function parameters did not change during or after the ultrasound examination. The LVAD flow was on average 4.3 ± 0.3 L/min, and the speed was 5247 ± 109 rotations/min. There were no signs that the LVAD caused substantial destruction of the ultrasound contrast agent; a regular dose of contrast agent could be used in all cases.

**Fig. 1:**
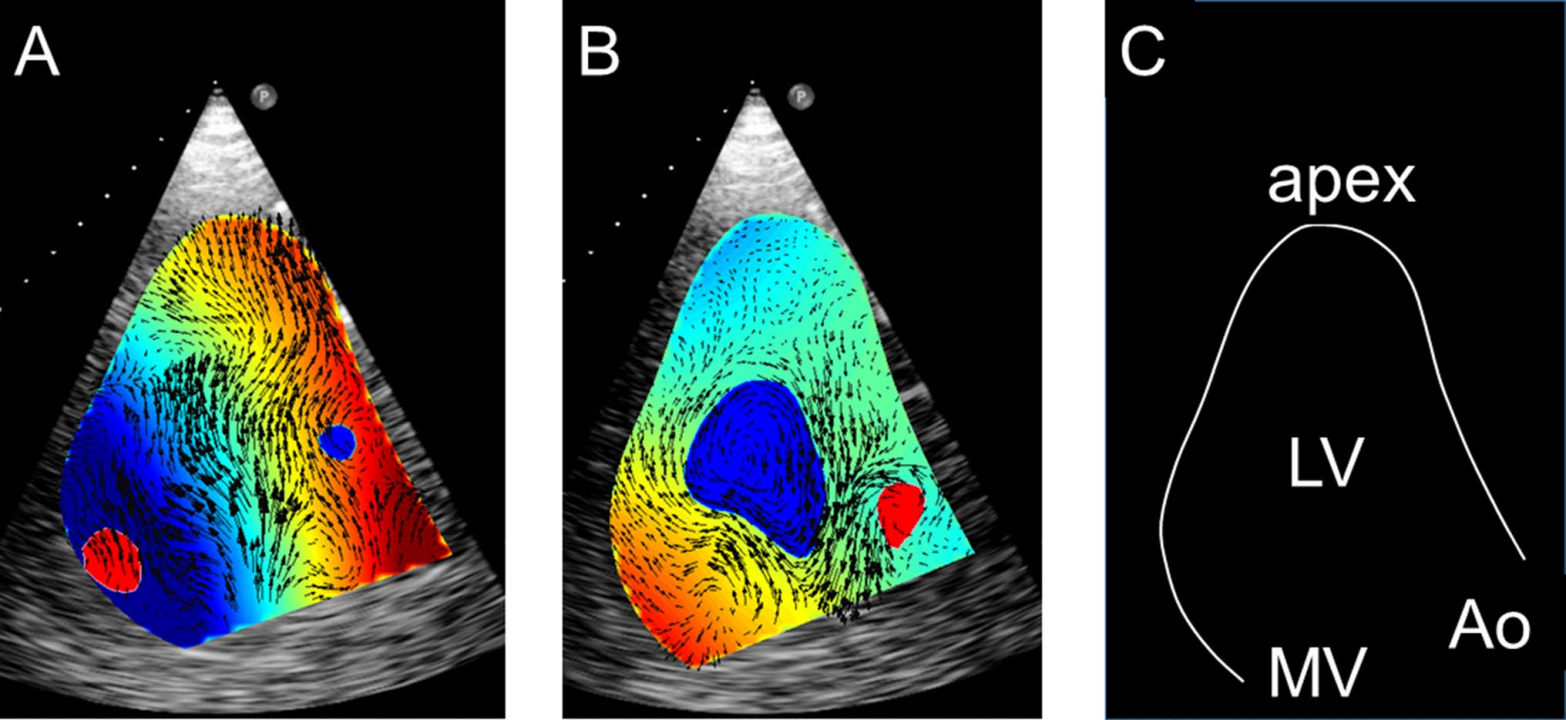
Example of LV intraventricular flow analysis using echo particle velocimetry in a patient with an LVAD (three-chamber apical view). **a** Demonstrates the intraventricular flow during diastole, with blood entering the LV, showing the LV blood flow toward the apex, where the LVAD inflow cannula is positioned. During systole (**b**), the intraventricular flow is directed toward the left ventricular outflow tract (LVOT). A clockwise intraventricular vortex is demonstrated in blue. **c** Demonstrates the position of the left ventricle (LV), left ventricular apex, mitral valve (MV), and aorta (Ao)

The results of the LV intraventricular flow quantification are presented in Table 2. Overall, substantial impairment of vortex parameters was observed in comparison with previously published normal values [9, 10]. The energy dissipation, vorticity, and kinetic energy fluctuation indices were severely impaired. Figure 1 provides an example of LV intraventricular flow analysis in a patient with an LVAD.

**Table 2:** Intraventricular flow quantification (*n* = 17)

	Measured value
Vortex area	0.30 ± 0.09
Vortex length	0.65 ± 0.17
Vortex depth	0.45 ± 0.08
Vortex intensity	− 0.36 ± 0.12
Energy dissipation index	0.96 ± 0.57
Vortex fluctuation index	0.75 ± 0.15
Kinetic energy fluctuation index	1.34 ± 0.46

Data are presented as mean ± standard deviation

## Discussion

This study demonstrates that LV intraventricular flow quantification with contrast-enhanced echocardiography in patients with an LVAD is safe and feasible. The main findings are that LV flow and vortex parameters are severely impaired in these patients. The observed intraventricular flow abnormalities are probably the result of pathophysiologic changes related to poor LV function and alterations in LV size and shape. Moreover, the LVAD inflow cannula is positioned near the LV apex, which affects the intraventricular flow and vortex formation. Previous observations have suggested that one of the benefits of an LV vortex is that it can minimize kinetic energy dissipation and reduce the total energy that is needed for LV ejection, resulting in more efficient energy use during the cardiac cycle [4, 9, 10]. Impairment of the vortex area or vortex intensity, which was observed in patients with poor LV function, may affect intraventricular flow efficiency, eventually leading to further deterioration in LV function and adverse LV remodeling.

In this study, the quantification of LV flow was performed by offline analysis of contrast-enhanced echocardiography using a previously validated nonvendor-specific software package called Hyperflow (Tomtec imaging systems Gmbh, Unterschleissheim, Germany). This echo particle image velocimetry software has good accuracy for the direction of flow and the quantification of low flow velocities, such as intraventricular flow [9, 10]. A limitation of this software is the estimation of higher flow velocities, such as the flow rate during peak velocity and the flow rate near the heart valves, which are underestimated because of the relatively low frame rate during image acquisition [9, 10]. However, this limitation is probably less relevant in patients with an LVAD because intraventricular flows are expected to be in the low-velocity range.

Agati et al. [9] studied intraventricular blood flow using quantitative echo particle image velocimetry in 34 consecutive patients with an ST elevation myocardial infarction (STEMI) and 30 healthy control subjects. The highest energy dissipation was observed in STEMI patients with a preserved global LV function, suggesting a fluid–tissue dynamical balance as a compensatory mechanism for maintaining adequate LV function. In patients with STEMI and a significantly reduced LV function, energy dissipation was reduced as a consequence of a low flow kinetic energy.

Cimino et al. [10] studied 32 patients with dilated cardiomyopathy who underwent cardiac resynchronization therapy (CRT). All patients underwent echo particle velocimetry 6 months after CRT during active CRT and during a temporarily discontinued CRT state. Energy dissipation, vortex area, and vorticity fluctuation were significantly higher in nonresponder patients during a temporarily discontinued CRT. During active CRT, these flow parameters further deteriorated in nonresponder patients.

The patient population in these previous studies [9, 10] differs from the current population of patients in that all our patients had advanced heart failure and poor LV function for which they had received an LVAD; these factors certainly have an influence on LV intraventricular flow dynamics. The methodology in the previous studies was similar. Both used contrast-enhanced echocardiography with the same ultrasound-enhancing agent (SonoVue, Bracco, Milan, Italy), and the same software package (Hyperflow, Tomtec imaging systems Gmbh, Unterschleissheim, Germany) was used for LV flow quantification as in the current study. The findings of the current study are in line with those of the previous two studies and show significant impairment of LV flow and vortex parameters in patients with poor LV function.

In patients with advanced heart failure due to poor LV function, LVAD therapy may significantly improve symptoms and prolong life expectancy [1–3]. However, several complications of LVAD therapy have been recognized, including LV thrombus formation, which may result in thromboembolic events such as stroke [1–3]. The present study demonstrated that a subgroup of patients with LVAD (four out of 17, 24%) had no LV output, and echo particle image velocimetry showed that in this subgroup, vortex area and vortex intensity were most severely affected. Further studies are needed to assess whether this subgroup has an increased risk of thromboembolic complications during follow-up. Subsequently, strategies such as an intensified anticoagulation treatment or a modification of LVAD settings can be evaluated in an attempt to reduce the risk of thrombosis.

This study showed, for the first time, that echo particle velocimetry allows the visualization of LV flow in patients with an LVAD. The main observation is that the physiological intraventricular flow and vortex formation are severely affected in these patients. In future studies, this method can be used as a tool to optimize intraventricular flow, possibly minimizing energy consumption during systole. Several modifiable factors may influence the intraventricular flow in patients with an LVAD, such as the exact implantation site of the LV cannula, the LVAD settings, and the LV filling state. Furthermore, additional factors, such as concomitant valve disease, atrioventricular and intraventricular conduction abnormalities, fluid balance, and systemic blood pressure, can be amendable factors for optimizing the intraventricular flow in these patients.

This study demonstrates that echo particle velocimetry is feasible and provides meaningful results, but it has several limitations. First, the study population was small because this was a prospective pilot study. Second, the presence of the LVAD hindered the acquisition of standard echocardiographic views in some patients because the inflow cannula was located near the LV apex. This limitation was overcome by a slightly modified transducer position, which may have resulted in marginally off-axis apical views. Third, the software package has good accuracy for the assessment of flow direction and the quantification of low flow velocities, but it underestimates high flow velocities [4, 9, 10]. Finally, no extensive safety data on the use of ultrasound-enhancing agents in patients with an LVAD are available. Recent small studies indicate that the use of ultrasound-enhancing agents in these patients is safe and results in improved image quality and a better delineation of endocardial borders [13, 14]. Contrast-enhanced ultrasound also has the potential to reveal rare vascular complications [15].

## Conclusion

Echo particle velocimetry is safe and feasible for the quantitative assessment of intraventricular flow in patients who have received an LVAD because of advanced heart failure and poor LV function. The intraventricular LV flow and vortex parameters are severely impaired in these patients.
